# Role and limitation of neoadjuvant hepatic arterial infusion chemotherapy in advanced hepatocelluar carcinoma patients with Child-Pugh class A

**DOI:** 10.1186/s12957-019-1685-6

**Published:** 2019-08-15

**Authors:** Beom-Hui Lee, Dong-Shik Lee, Chan Woo Cho, Sung-Su Yun

**Affiliations:** Department of Surgery, College of Medicine, Yeungnam University, 170 Hyeonchungno, Nam-gu, Daegu, 42415 Republic of Korea

**Keywords:** Hepatocellular carcinoma, Liver resection, Neoadjuvant hepatic arterial infusion chemotherapy

## Abstract

**Background:**

Patients with advanced hepatocellular carcinoma (HCC) have a poor oncologic outcome. In this study, we evaluated the role and limitation of neoadjuvant hepatic arterial infusion chemotherapy (HAIC) in advanced HCC patients with Child-Pugh class A and the efficacy of liver resection subsequent to downstaging after neoadjuvant HAIC.

**Methods:**

In the present retrospective study, 103 patients with advanced HCC, who underwent neoadjuvant HAIC from April 2003 to March 2015 were analyzed. Response to HAIC was evaluated by dividing time period into after 3 cycles and after 6 cycles, each defined as early and late period. Liver resection after neoadjuvant HAIC was offered in patients who were considered as possible candidates for curative resection with tumor-free margin as well as sufficient future liver remnant volume.

**Results:**

The median survival time (MST) in all patients was 14 ± 1.7 months. Response rate and disease control rate were 36.3% (37) and 81.4% (83) in early period, respectively, and 26.4% (14) and 47.2% (25), in late period, respectively (*P* = 0.028). Twelve patients (11.7%) underwent liver resection after neoadjuvant HAIC and the MST was 37 ± 6.6 months. One-, 3-, and 5-year recurrence-free survival after liver resection were 58.3%, 36.5%, and 24.3% respectively. Liver resection was identified as the only independent prognostic factor that associated with overall survival in multivariate analysis (*P* = 0.002)

**Conclusion:**

HAIC could be further alternative for the treatment of advanced HCC in patients with good liver function. If liver resection is possible after neoadjuvant HAIC, liver resection would provide better outcomes than HAIC alone.

## Introduction

There exist different opinions between surgeons and hepatologists in Eastern and Western countries, especially in the treatment of advanced hepatocellular carcinoma (HCC). Results of resection, transplantation, and systemic chemotherapy are not satisfactory, and recent trials of target therapies such as sorafenib and regorafenib in Western countries also are not so effective, despite initial expectations [1–4]. In patients with advanced HCC, the prognosis is extremely poor and median survival time (MST) is approximately 2.7–7 months [5, 6].

We tried neoadjuvant hepatic arterial infusion chemotherapy (HAIC) in patients with advanced HCC and good liver function; most of the patients had multiple bilobar tumors and tumor(s) with main portal vein invasion.

We initiated HAIC based on the pharmacological benefits compared to that of intravenous injection. After intravenous injection, the chemotherapeutic drug reaches to the heart and only 5% of the cardiac output goes through the hepatic artery. Furthermore, most of the drugs lose more than 50% of their efficacy after 1 cycle of systemic circulation [7–10]. On the other hand, HAIC is more effective than intravenous chemotherapy because of its first-pass metabolism and topical accumulation of chemotherapeutic agents in the liver [7, 11, 12]. Fortunately, it is already demonstrated that HAIC in patients with advanced HCC resulted in the favorable response rate (RR) and survival benefits [5, 9, 13, 14].

In this study, we evaluated the role and limitation of neoadjuvant HAIC in advanced HCC patients with Child-Pugh class A and the efficacy of liver resection subsequent to downstaging after HAIC.

## Methods

### Patients

From April 2003 to March 2015, 136 patients with advanced HCC underwent neoadjuvant HAIC at our institution. HAIC was performed in preserved functional liver reserved with Child-Pugh class A and in patients with advanced HCC according to the Barcelona Clinic Liver Cancer guideline. In this retrospective study, patients with serious comorbidities such as cardiopulmonary insufficiency or other medical condition, or concurrent malignant tumor were excluded. Patients who did not undergo more than 3 cycles of HAIC were also excluded. After exclusion, a total of 103 patients was analyzed in this study. This study was approved by the Institutional Review Board of Yeungnam University Medical Center, Daegu, Republic of Korea (IRB no. 2018-12-016-001).

### Treatment protocol

HAIC was performed via a port system inserted through a femoral artery in a subcutaneous pocket in the right thigh. The patients were treated with 5-fluorouracil (5-FU, JW Pharmaceutical, Seoul, Korea, 750 mg/m^2^) for 2 h and cisplatin (JW Pharmaceutical, Seoul, Korea, 25 mg/m^2^) for 1 h from day 1 to 4. Chemotherapeutic agents were repeatedly administrated every 4 weeks after evaluating the adverse effects of HAIC. Intravenous hydration with antiemetic treatment was performed before and after cisplatin infusion to prevent cisplatin-induced nephrotoxicity.

The response to HAIC was evaluated after 3 and 6 cycles. The early period response was defined as after 3 cycles of HAIC, and the late period response was defined as after 6 cycles of HAIC. The tumor response was evaluated by contrast-enhanced computed tomography and tumor marker after every 3 cycles of HAIC. Radiologic response to treatment was assessed by Modified Response Evaluation Criteria in solid tumors and classified as complete response (CR), partial response (PR), stable disease (SD), or progressive disease (PD). The response was defined as the achievement of CR or PR and the disease control was defined as the achievement of CR, PR, or SD. To evaluate biologic tumor response, the alterations in alpha-fetoprotein (AFP) were analyzed based on initial AFP value and AFP value after treatment.

The operability was assessed through multidisciplinary discussions with hepatobiliary surgeon, hepatologist, and radiologist. Liver resection was offered to patients who were considered as the candidates for curative resection with tumor-free margin as well as sufficient future liver remnant volume.

### Statistical analysis

Continuous variables were expressed as mean with standard deviation. Categorical variables were calculated using the chi-square test and Fisher’s exact test. Survival was calculated using the Kaplan-Meier method, and differences between the groups were compared using the log-rank test. Variables associated with survival were evaluated by univariate and multivariate analyses using a Cox proportional hazard model. All statistical analyses were performed using statistical software IBM SPSS version 21.0 (IBM Co., Armonk, NY, USA). Statistical significance was defined as a *P* value of less than 0.05.

## Results

### Baseline characteristics of the patients

The baseline characteristics of the patients are summarized in Table 1. The mean age of the patients was 53.7 ± 9.0 years and 93 patients (90.7%) were male. The most common etiology for HCC was hepatitis B virus (77.7%) and the second was alcohol (7.7%). The number of patients with bilobar HCC or portal vein tumor thrombus (PVTT) was 59 (57.3%) and 69 (67.0%) respectively. Extrahepatic metastasis was identified in 20 (19.4%) patients at the time of enrollment, and lung or lymph nodes were predominant.

**Table 1:** Baseline characteristics of patients treated with HAIC for advanced hepatocellular carcinoma

Characteristic	
Mean age (years)	53.7 ± 9.0
Gender, male	93 (90.3%)
Etiology	
HBV	80 (77.7%)
HCV	4 (3.9%)
Alcohol	8 (7.7%)
Others	11 (10.7%)
Preoperative laboratory test	
Total bilirubin (mg/dL)	1.1 ± 0.6
PT (%)	92.7 ± 16.0
Albumin (g/dL)	3.9 ± 0.5
Platelet count (×10^3^/μL)	184.5 ± 89.4
AFP (ng/mL)	10587.7 ± 25449.0
PIVKA-II (mAU/mL)	7386.3 ± 19924.9
Tumor size (mm)	70.9 ± 39.8
Tumor number	
Single	19 (18.4%)
Multiple	84 (81.6%)
Bilobar HCC	59 (57.3%)
PVTT	69 (67.0%)
Extrahepatic metastasis	20 (19.4%)
Lung	8 (7.7%)
Lymph node	9 (8.7%)
Lung and lymph node	1 (1.0%)
Bone	1 (1.0%)
Adrenal gland	1 (1.0%)
HAIC cycle	5.7 ± 2.3
Previous locoregional treatment	35 (34.0%)

*HAIC* hepatic arterial infusion chemotherapy, *SD* standard deviation, *HBV* hepatitis B, *HCV* hepatitis C, *PT* prothrombin time, *AFP* alpha-fetoprotein, *PIVKA-II* proteins induced by vitamin K antagonist-II, *HCC* hepatocellular carcinoma, *PVTT* portal vein tumor thrombus

### Comparison of response to HAIC between the early and late period

MST in the patients treated with HAIC was 14 ± 1.7 months. Patients who obtained CR, PR, SD, and PD were 2 (2.0%), 35 (34.3%), 46 (45.1%), and 19 (18.6%) in early period, respectively, and 5 (9.4%), 9 (17.0%), 11 (20.8%), and 28 (52.8%) in late period, respectively. In the early period, RR and disease control rate (DCR) were 36.3% (37) and 81.4% (83), respectively. RR and DCR in the late period were 26.4% (14) and 47.2% (25), respectively. RR in the early period to HAIC was significantly better compared to the late period (*P* = 0.028, Table 2).

**Table 2:** Comparison of response between the early and late period response

Response	Early period^a^ (after 3 cycles)	Late period^b^ (after 6 cycles)	*P* value
CR	2 (2.0%)	5 (9.4%)	
PR	35 (34.3%)	9 (17.0%)	
SD	46 (45.1%)	11 (20.8%)	
PD	19 (18.6%)	28 (52.8%)	
RR (CR + PR)	37 (36.3%)	14 (26.4%)	0.028
DCR (CR + PR + SD)	83 (81.4%)	25 (47.2%)	0.492

*CR* complete response, *PR* partial response, *SD* stable disease, *PD* progressive disease, *RR* response rate, *DCR* disease control rate

^a^One patient was excluded because there was no sufficient data for evaluating response to treatment

^b^The late period response was evaluated in 53 patients

### Liver resection subsequent to downstaging after neoadjuvant HAIC

**Fig. 1:**
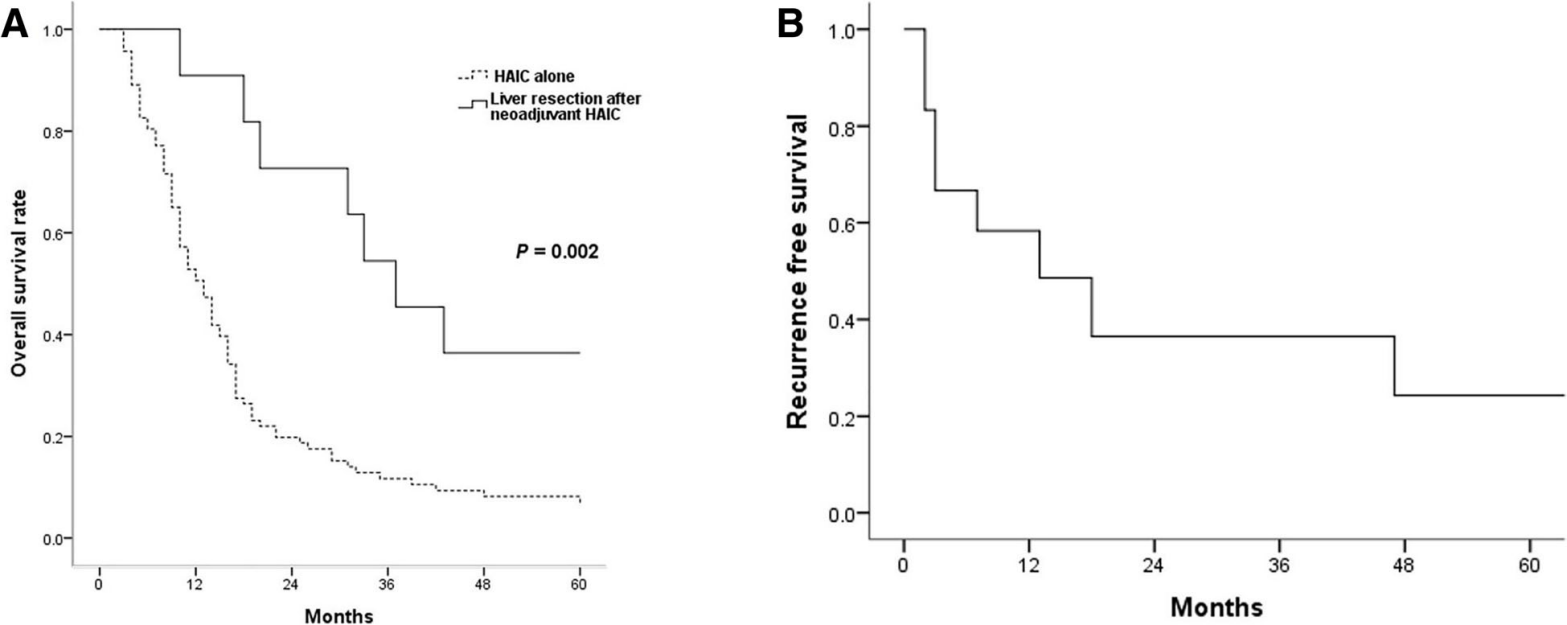
**a** Overall survival between liver resection after neoadjuvant HAIC and HAIC alone group. **b** Recurrence-free survival rate in liver resection after neoadjuvant HAIC group. *HAIC* hepatic arterial infusion chemotherapy

Twelve (11.7%) patients underwent liver resection after neoadjuvant HAIC. In liver resection after neoadjuvant HAIC group, MST and overall survival (OS) were significantly better compared to HAIC alone group (MST 37 ± 6.6 vs. 13 ± 1.4 months, *P* = 0.002, Fig. 1a). One-, 3-, and 5-year recurrence-free survival (RFS) were 58.3%, 36.5%, and 24.3% respectively (Fig. 1b). The median time to recurrence was 13 ± 7.4 months. Five patients had bilobar HCC, 10 patients had PVTT, and 1 patient had both bilobar HCC and PVTT. Extrahepatic metastasis was found in 1 patient and identified as paraaortic lymph node metastasis, and complete remission of the metastatic lesion after HAIC was recognized. Tumor size reduction and PVTT regression were the main causes of liver resection. The recurrence was identified in 10 patients, and intrahepatic recurrence in 7 patients, systemic recurrence in 2 patients, and both in 1 patient. Details of the patients were summarized in Table 3.

**Table 3:** Profiles of patients who underwent liver resection after neoadjuvant HAIC

Case	Gender/age	Etiology	HAIC cycle	AFP (initial-preoperative)	Bilobar HCC	PVTT	Extrahepatic metastasis	Resection	Margin	Recurrence (time/site) (months)	Survival time (months)
1	F/73	HBV	6	3170–99.4	No	Yes	Yes (LNs)	Minor	Tumor-free	No	81
2	M/44	HBV	6	5845–2.8	No	Yes	No	Major	Tumor-free	No	120
3	M/61	HBV	8	11.74–8.8	No	Yes	No	Major	Tumor-free	18/lung	46
4	M/40	HBV	4	158.0–432.2	No	Yes	No	Major	Tumor-free	2/liver	18
5	M/42	HBV	6	950–60.2	No	Yes	No	Major	Tumor-free	13/lung	31
6	M/53	HBV	4	369.9–337.7	No	Yes	No	Major	Tumor-free	2/liver	10
7	F/48	HBV	6	188663–40762	Yes	No	No	Minor	Tumor-free	3/liver	37
8	M/44	HBV	7	7.5–6.7	Yes	Yes	No	Minor	Tumor-free	10/liver	33
9	M/56	HBV	3	911.6–23.1	No	Yes	No	Major	Tumor-free	47/liver	68
10	M/47	HBV	6	1038–701.1	Yes	No	No	Major	Tumor-free	13/liver, lung	43
11	F/39	HBV	8	121000–6047	Yes	Yes	No	Major	Tumor-involved	7/liver	20
12	M/46	HBV	6	188.6–17.2	Yes	Yes	No	Minor	Tumor-free	3/liver	67

*HAIC* hepatic arterial infusion chemotherapy, *HBV* hepatitis B, *AFP* alpha-fetoprotein, *HCC* hepatocellular carcinoma, *PVTT* portal vein tumor thrombus, *LN* lymph node

### Prognostic factors associated with overall survival of HAIC

In univariate analysis, OS was associated with total bilirubin level and liver resection. While liver resection was the only independent prognostic factor to be associated with OS in multivariate analysis (hazard ratio, 3.480; 95% confidence interval, 1.587–7.630; *P* = 0.002, Table 4).

**Table 4:** Prognostic factors associated with overall survival of HAIC

Risk factors	Univariate	Multivariate	
	*P* value	HR (95% CI)	*P* value
Age (years)	0.123		
Gender	0.870		
Total bilirubin (mg/dL)	0.033	1.312 (0.943–1.825)	0.108
Platelet (× 10^3^/mL)	0.342		
Albumin (g/dL)	0.556		
PT (%)	0.153		
AFP (ng/mL)	0.446		
PIVKA-II (m AU/mL)	0.205		
Tumor size	0.446		
Tumor number	0.105		
Previous locoregional treatment	0.528		
PVTT	0.407		
Extrahepatic metastasis	0.595		
HAIC cycle	0.119		
Liver resection after HAIC	0.001	3.480 (1.587-7.630)	0.002

*HAIC* hepatic arterial infusion chemotherapy, *HR* hazard ratio, CI, confidence interval, *PT* prothrombin time, *AFP* alpha-fetoprotein, *PIVKA-II* proteins induced by vitamin K antagonist-II, *PVTT* portal vein tumor thrombus, *ORR* objective response rate

## Discussion

There are a few remaining treatment options for patients with advanced HCC. Sorafenib is regarded as the first line therapy for advanced HCC according to BCLC guideline and other molecular target agents such as lenvatinib or regorafenib are considered as effective alternatives [3]. However, MST and RR in patients who treated with sorafenib is unsatisfactory (10.7 months and 2 to 3.3%, respectively) [4, 15]. In REFLECT trial, the efficacy of lenvatinib exhibited a limitation because patients with major PVTT and with more than 50% of liver involvement were excluded [3]. In this regard, HAIC is considered as an effective alternative with better RR than sorafenib. There exist a number of studies associated with HAIC in East Asia stating MST and RR in patients treated with HAIC as 6.5 to 14 months and 24 to 52%, respectively [7, 8, 16–18]. In our study, MST and RR were 14 ± 1.7 months and 26.4%, respectively, with values consistent with previous studies.

Liver resection for advanced HCC is still controversial because of high surgical risk, poor prognosis, and difficulty in judging operability although several studies have shown a survival benefit in intermediate or advanced HCC [19–24]. On the contrary, downstaging after neoadjuvant HAIC provides an opportunity for the patients who undergo liver resection to obtain better survival outcomes. In our study, 12 patients (11.7%) who underwent liver resection after neoadjuvant HAIC had better OS than patients who underwent HAIC alone. One patient had a postoperative complication of grade III or higher because of postoperative bleeding. However, there were no postoperative morbidity and mortality associated with poor liver function. Several studies have reported survival benefits of liver resection after downstaging using neoadjuvant HAIC or HAIC with radiotherapy [16, 25, 26] and exhibited similar results. Considering these acceptable results, liver resection subsequent to downstaging after neoadjuvant treatment can be regarded as one of the treatment options for patients with advanced HCC and with good liver function. To obtain downstaging of HCC, it is necessary to know whether there is a response to treatment or not as early as possible. In our study, radiologic findings and tumor marker were used for evaluation of response to HAIC after every 3 cycles. In addition, the alteration in AFP level was used for early assessment of response so that we could predict tumor burden and biology. In early period, RR was significantly better than compared to late period (36.3% vs. 26.4%, *P* = 0.028). It was considered that the resistance to HAIC was caused by repeated HAIC. Thus, in patients with both a radiologic response and decreased or normalized tumor markers in the early period, we recommend repeat evaluation resectability as early as possible. And if possible, liver resection should be considered to obtain survival benefits before progression of HCC or resistance to HAIC. On the contrary, in case of increase in AFP value during the treatment, precise evaluation of response to HAIC should be carried out. And if resistance to HAIC is predicted, switching to other treatments should be considered at an early period.

In several studies, 1- and 5-year RFS after hepatectomy in HCC patients were reported to range from 65 to 72% and from 30 to 39%, respectively. The median time to recurrence was 22 to 34 months [27, 28]. Although our study showed worse outcomes than previous studies, we hypothesize that our results could be acceptable while considering the fact that we analyzed patients with advanced HCC subsequent to downstaging after neoadjuvant HAIC.

In this study, patients with extrahepatic metastasis were included. Although HAIC with focus on intrahepatic lesions and extrahepatic metastasis has not responded well, intrahepatic lesions were considered as a significant prognostic factor in survival rather than extrahepatic metastasis in previous studies [7, 29, 30]. In the present study, extrahepatic metastasis was not identified as a significant factor in OS. Thus, it is proposed that the use of HAIC could be considered in patients with extrahepatic metastasis.

One of the limitations of this study was that there was no consensus for the standard treatment protocol of HAIC. A number of studies have reported various treatment regimen for HAIC. Most commonly used regimens are 5-fluorouracil with low-dose cisplatin (FP), and others are cisplatin alone, high-dose FP, FP plus interferon, FP plus leucovorin and FP plus epirubicin [3, 7–9, 13, 14, 31]. We designed a treatment protocol using low-dose FP. However, the optimal regimen for HAIC still remains a controversial issue. In addition, the included data from a single institution were retrospectively analyzed; hence, we could not completely exclude the selection-bias and could not obtain a sufficient number of cases. Apparently, though it is difficult to conduct randomized controlled trials for HAIC because of ethical issues, further investigations for optimization of HAIC protocol and a large-scale multicenter study are needed.

## Conclusion

HAIC could be another alternative for the treatment of advanced HCC in patients with good liver function. If liver resection is possible after neoadjuvant HAIC, liver resection would provide better outcomes than HAIC alone.

## Data Availability

The anonymized data used and/or analyzed during the current study are available from the corresponding author on reasonable request.

## Abbreviations

AFP: Alpha-fetoprotein
CR: Complete response
DCR: Disease control rate
FP: 5-fluorouracil with cisplatin
HAIC: Hepatic arterial infusion chemotherapy
HCC: Hepatocellular carcinoma
MST: Median survival time
OS: Overall survival
PD: Progressive disease
PR: Partial response
PVTT: Portal vein tumor thrombus
RFS: Recurrence-free survival
RR: Response rate
SD: Stable disease
